# A Diagnostic Gene-Expression Signature in Fibroblasts of Amyotrophic Lateral Sclerosis

**DOI:** 10.3390/cells12141884

**Published:** 2023-07-18

**Authors:** Giovanna Morello, Valentina La Cognata, Maria Guarnaccia, Vincenzo La Bella, Francesca Luisa Conforti, Sebastiano Cavallaro

**Affiliations:** 1 Institute for Biomedical Research and Innovation, National Research Council (CNR-IRIB), 95126 Catania, Italy; giovanna.morello@irib.cnr.it (G.M.); valentina.lacognata@cnr.it (V.L.C.); maria.guarnaccia@cnr.it (M.G.); 2 ALS Clinical Research Center and Neurochemistry Laboratory, BiND, University of Palermo, 90133 Palermo, Italy; vincenzo.labella@unipa.it; 3 Medical Genetics Laboratory, Department of Pharmacy and Health and Nutritional Sciences, University of Calabria, 87036 Rende, Italy; francescaluisa.conforti@unical.it

**Keywords:** amyotrophic lateral sclerosis, transcriptomics, network, machine learning, molecular signature, class prediction, disease diagnosis

## Abstract

Amyotrophic lateral sclerosis (ALS) is a fatal, progressive neurodegenerative disease with limited treatment options. Diagnosis can be difficult due to the heterogeneity and non-specific nature of the initial symptoms, resulting in delays that compromise prompt access to effective therapeutic strategies. Transcriptome profiling of patient-derived peripheral cells represents a valuable benchmark in overcoming such challenges, providing the opportunity to identify molecular diagnostic signatures. In this study, we characterized transcriptome changes in skin fibroblasts of sporadic ALS patients (sALS) and controls and evaluated their utility as a molecular classifier for ALS diagnosis. Our analysis identified 277 differentially expressed transcripts predominantly involved in transcriptional regulation, synaptic transmission, and the inflammatory response. A support vector machine classifier based on this 277-gene signature was developed to discriminate patients with sALS from controls, showing significant predictive power in both the discovery dataset and in six independent publicly available gene expression datasets obtained from different sALS tissue/cell samples. Taken together, our findings support the utility of transcriptional signatures in peripheral cells as valuable biomarkers for the diagnosis of ALS.

## 1. Introduction

Amyotrophic lateral sclerosis (ALS) is a heterogenous neurodegenerative disorder classically defined by the loss of upper and lower motor neurons, resulting in rapidly progressive paralysis and respiratory insufficiency within three to five years after the symptoms begin [1]. The clinical manifestations of ALS are variable in terms of age and site of onset, relative degree of upper and lower motor neuron involvement, rate of progression of symptoms, and the occurrence of cognitive and behavioral changes. ALS can be familial (fALS), defined by its presence in more than one family member and accounting for 10–15% of known cases, or sporadic (sALS), accounting for 85–90% of cases with no clear genetic family history and probably associated with a polygenic and multifactorial etiology [1]. During the past years, a rapidly increasing number of genetic risk factors have been identified, and multiple biological processes have been linked to ALS, including RNA processing, excitotoxicity, oxidative stress, cytoskeletal abnormalities, impaired axonal transport, neuroinflammation, mitochondrial dysfunction, and protein aggregation [2].

Despite increasing recognition of genetic and pathological contributions, the underlying causes of ALS remain poorly understood, and important questions in clinical practice still have to be answered. Except for Riluzole and Edaravone, which provide only modest clinical benefits, there is a dearth of effective disease-modifying therapies in ALS [3]. In this setting, the lack of ALS biomarkers to enable an accurate diagnosis and monitoring of disease progression is a limiting factor for the identification, development, and testing of new drug candidates. In the absence of a definite diagnostic test, the diagnosis of ALS is based on clinical and electrophysiological examination as well as medical history and the exclusion of diseases mimicking ALS, as set out by a range of diagnostic criteria: El Escorial [4], Airlie House [5], Awaji [6], and Gold Coast [7]. However, this classification system is often found to be inadequate due to the heterogeneity and non-specific nature of the initial symptoms of ALS, leading to a diagnostic delay of 9–15 months from onset to diagnostic confirmation, with dire consequences in a relentlessly progressive disorder where prompt therapeutic intervention is crucial. Therefore, elucidating complex mechanisms underlying sALS and identifying new reliable biomarkers represents an urgent need to improve diagnostic speed and accuracy and provide the basis for the development of effective therapeutics [8].

Over the last two decades, intensive work has been carried out to find consistent clinical biomarkers for ALS [9,10,11,12,13]. In particular, gene expression studies have been demonstrated to be powerful in providing valuable insights into the molecular basis underlying ALS pathophysiology and identifying molecular signatures or biomarkers able to classify ALS patients into selective clinically relevant subtypes characterized by different biological properties, prognostic biomarkers, and treatment options [14,15,16,17,18,19]. Within this context, the emerging use of machine learning approaches to find genetic biomarkers or construct robust disease classifiers based on patients’ gene expression data is revolutionizing clinical decision-making in multiple complex human conditions, including cancer and cardiovascular diseases, and proving to be an exciting tool and promising option for hopefully improving our skills also in neurological conditions [20,21,22,23,24,25,26,27,28].

In the last few years, our research group and others have established an important foundation for the molecular diagnosis and taxonomy of ALS by using postmortem cortex transcriptomics to discriminate between controls and sALS patients and stratifying these latter into distinct molecular subtypes [9,19,29,30,31,32,33,34,35,36,37,38,39,40,41,42,43,44,45]. However, while the analysis of *post-mortem* brain samples allows for the acquisition of relevant information on disease mechanisms and potential drug targets, it is not readily useful for diagnostics. On the other hand, although not primarily affected by the disease, peripheral cells of ALS patients—such as blood cells and fibroblasts—may represent a valuable source for diagnostic ‘signatures’, since they are readily obtainable from living donors, retain the genetic background of the patient, and share some of the pathological features found in the central nervous system (CNS) [14,15,46,47]. To this regard, despite the fact that blood-based tissues (lymphocytes) are more readily obtained, in vitro expansion of fibroblasts is significantly easier given the robustness of these cells in tissue culture, making them a more practical, cost-effective, easy, and fast established disease model.

In this study, we identify a transcriptome-based signature in sALS fibroblasts that may be used as a molecular classifier to discriminate between ALS and control individuals. In particular, we analyzed the whole gene expression profiles of skin fibroblasts from sALS patients and healthy controls. Differentially expressed genes were then used to build a machine-learning-based classifier for discriminating patients with ALS from controls. To validate the reliability and accuracy of this transcriptome signature, class prediction was also performed in different independent publicly available ALS transcriptomic datasets from various sources, including skin fibroblasts, whole blood, and post-mortem central tissues [14,15,40,46,47,48].

## 2. Materials and Methods

### 2.1. Subjects

With written informed consent, fibroblast samples were obtained from three healthy individuals and nine patients diagnosed with sALS. All patients, diagnosed according to the El Escorial revised criteria [49], were previously screened for the presence of pathogenic mutations in the *C9ORF72*, *SOD1*, *ANG*, *FUS*, and *TARDBP* genes and showed no mutation [44]. The average age at the time of skin biopsy in healthy controls and ALS fibroblast cases was 64 years (range 56–72 years) and 64 years (range 47–79 years), respectively. The median time from onset of ALS symptoms to biopsy was 12 months, with an average age of 63 at the time of diagnosis. We scored the functional decline of the disease through the revised ALS Functional Rating Scale (ALSFRS-R) [50] and used the ∆FS (ALSFRS-R at onset-ALSFRS-R at time of diagnosis/diagnostic delay) to derive the rate of progression [51]. Three different rates of progression can therefore be identified: slow (∆FS < 0.5), intermediate (∆FS ≥ 0.5 < 1), rapid (∆FS ≥ 1). In our cohort of nine sALS patients, the median ∆FS was 0.52 (IQR = 0.39–1.11), suggesting a slow-intermediate progression. All patients were Caucasian and were recruited from a single ALS Center in Palermo, Italy. A summary of the disease characteristics and demographics of all subjects enrolled in this study is shown in Table 1. The University of Palermo Review Board approved this consent procedure and the entire study (document 04/2019), and the participants signed informed consent prior to the study.

### 2.2. Primary Fibroblast Isolation and Culture

Primary fibroblast lines were established from punch skin biopsy samples, as previously described in detail [52], in accordance with guidelines set by the local ethics committee. Once established, primary fibroblast cultures were maintained in Dulbecco’s Modified Eagle Medium (DMEM) high glucose (Life Technologies, Carlsbad, CA, USA) medium supplemented with 10% calf serum, 2 mM L-glutamine, 5 mM pyruvate, 100 U/mL penicillin, and 100 μg/mL streptomycin. The medium was changed every 3–4 days until the fibroblasts were grown to confluence. Fibroblasts were maintained in culture through passages in a flask. All experiments were performed in confluent cells at the 3rd/4th passage on the flask.

### 2.3. RNA Isolation, Microarray Processing, and Data Extraction

Total RNA was extracted from fibroblasts by standardized protocols using TRIzol Reagent (Invitrogen, Carlsbad, CA, USA) according to the manufacturer’s guidelines. Total RNA was quantified on an Agilent 2100 Bioanalyzer (Agilent Technologies, Palo Alto, CA, USA), and samples with an RNA integrity score (RIN) higher than 9 were qualified for further processing. For microarray analysis, the Agilent array platform was used. Sample preparation and microarray hybridization were performed based on the manufacturer’s standard protocols, as previously described [53]. Briefly, 1 ug of total RNA from each sample was amplified and transcribed into fluorescent complementary DNA (cDNA) using the Low RNA Input Fluorescent Linear Amplification Kit (Agilent Technologies, Inc., CA, USA), after which labelled RNA was cleaned using RNeasy column purification (Qiagen, Venlo, the Netherlands). The Cyanine-3 (Cy3) labelled cRNA samples were hybridized onto the Whole Human Genome Oligo Microarray (4 × 44 K; Agilent Technologies, Inc., Santa Clara, CA, USA). Aliquots (750 ng) of Cy3 labeled cRNA targets were co-hybridized on 4 × 44 K Whole Human Genome Oligo Microarrays (Agilent Technologies, Italy). Microarray hybridization and washing were performed using reagents and instruments (hybridization chambers and a rotating oven) as indicated by the manufacturer (Agilent Technologies, Palo Alto, CA, USA). Arrays were then scanned at 3 µm resolution using an Agilent G4900DA SureScan Microarray Scanner System (Agilent Technologies, Palo Alto, CA, USA). Raw microarray data were acquired and analyzed using Agilent’s Feature Extraction v.12.1 software to assess the array spot quality as well as check signal and background intensity statistics in the default setting. Raw microarray data were deposited in NCBI’s Gene Expression Omnibus (GEO) with the accession number GSE233881.

### 2.4. Gene Expression Profiling and Class Prediction Modeling

Raw signal values were thresholded to 1, log2 transformed, normalized to the 75th percentile, and baselined to the median of all samples using GeneSpringGX v.14.9.1 (Agilent Technologies, Palo Alto, CA, USA). A moderate *t*-test followed by Benjamini and Hochberg’s False Discovery Rate (FDR) was applied to detect differential expression across sALS and healthy control groups. Transcripts were defined as differentially expressed if they differed between groups with a fold change (FC) of >2 fold and an FDR-corrected *p*-value of <0. Unsupervised hierarchical clustering of differentially expressed genes (DEGs) was performed using a Euclidean distance measure and Ward’s linkage rule in the GeneSpringGX program.

The identified DEGs were subjected to class prediction analysis in order to evaluate their ability to accurately classify patients into distinct clinical phenotypes based upon their expression profiles. Class prediction was performed using the machine learning algorithm ‘Support Vector Machines’ (SVM) of GeneSpring. The model was built using sample classifiers ‘sALS’ or ‘CTRL’ with the linear Kernel function (maximum iterations = 100,000, cost = 100, ratio = 1) and a leave-one-out cross-validation analysis. The SVM model was built in our study cohort (*training test*) and validated in six independent ALS datasets (*test sets*) to prevent over-fitting the predictive signature (Table 2). Test/validation sets were obtained from the Gene Expression Omnibus (https://www.ncbi.nlm.nih.gov/geo/; accessed on 19 May 2023) and ArrayExpress database (https://www.ebi.ac.uk/arrayexpress/experiments/E-TABM-940/; accessed on 19 May 2023) and included gene expression data from skin and peripheral nerve-fibroblasts (GSE56808, GSE68240), whole blood (GSE11280, GSE112676, E-TABM-940), and *post-mortem* motor cortex (E-MTAB-2325) samples of sALS patients and age-matched controls [14,15,40,46,47,48] (Table 2). Clinical and demographic details of patients and controls for each dataset are described in the original articles [14,15,40,46,47,48].

### 2.5. Functional Enrichment and Network Analysis

Functional properties of DEGs in sALS fibroblasts were evaluated by testing for enrichment of gene ontology (GO) and known biological pathway annotations using multiple databases and software (PANTHER, www.pantherdb.org; accessed on 12 May 2023); Reactome, http://reactome.org; Metacore, https://portal.genego.com; accessed on 12 May 2023). All these resources identified significantly enriched terms associated with a given list of genes by calculating the hypergeometric distribution. In particular, the GO (http://www.geneontology.org; accessed on 12 May 2023) database contains terms for the functional classification of genomic data under three main categories: biological processes (BP), cellular components (CC), and molecular functions (MF). The significance of GO terms and biological pathways was determined using the Fisher‘s exact test. *p*-value < 0.05 and gene counts > 2 were set as thresholds to filter out significant terms.

Next, functional interactions among proteins encoded by DEGs were analyzed by building a protein-protein interaction (PPI) network. In particular, the protein interaction data were selected from the Search Tool for the Retrieval of Interacting Genes/Proteins (STRING) database [54,55], and the resulting PPI network was visualized with the Cytoscape software (v.3.9.1), an open-source software for visualization, modelling and integration of biomolecular interaction networks [56]. In the PPI network, the protein is defined as the node, and the interaction between two nodes is defined as the edge. The extended network was constructed by using the DEGs as seed molecules and setting a high level of confidence between molecular interactions (a high confidence score of at least 0.8) and a maximum number of interactions to The significant hub nodes in the PPI network were selected according to the scoring of the maximum correlation criterion (MCC) by using the Cytoscape plugin cytoHubba [57], which explores important nodes and modules by topological algorithms. The topological parameter indicates the importance of a node (gene/protein) among the functionally connecting links in a PPI network. The top twenty genes scoring the highest in the PPI network were identified as hub genes in the present study. Subsequently, the Molecular Complex Detection (MCODE) plugin was applied to find highly connected clusters of genes in the PPI network with the following cut-off criteria: Degree cutoff = 2, node score cutoff = 0.2, k-core = 2, and max. depth = Identified clusters with more than five nodes and the selected hub genes were analyzed by the BinGO plug-in of Cytoscape [58] for functional enrichment analysis.

## 3. Results

### 3.1. Transcriptome Profiles Reveal a Molecular Signature for sALS Fibroblasts

**Figure 1:**
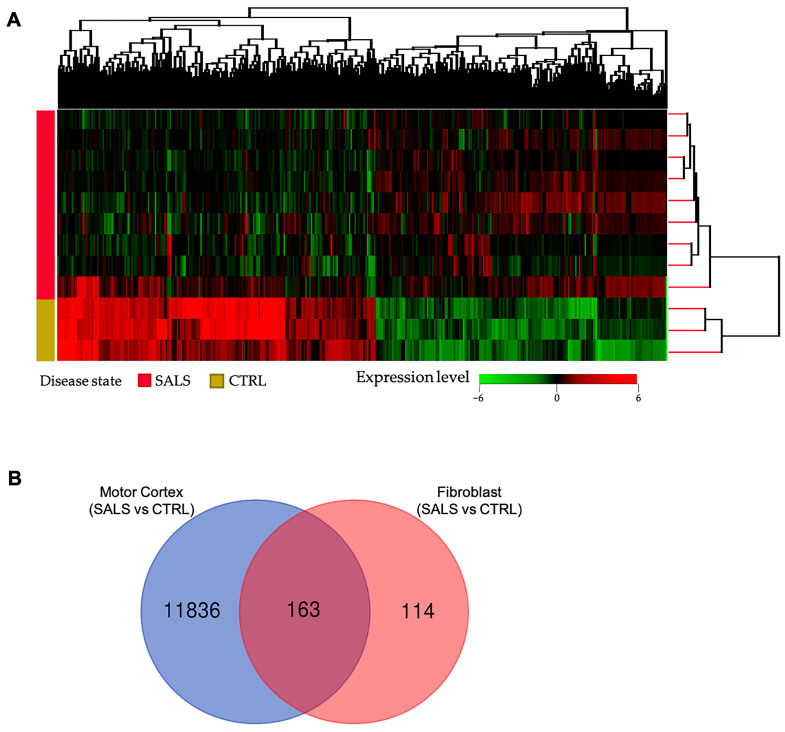
**Transcriptome analysis revealed molecular signatures for sALS fibroblasts.** (**A**) Unsupervised hierarchical clustering analysis (similarity measure: Euclidean; linkage rule: Wards) of 277 DEGs among sALS and control subjects. The heatmap shows the median-normalized expression of individual genes across all samples, where genes and patients were clustered on the basis of expression similarities. In this two-dimensional presentation, each row represents a single gene, and each column represents a fibroblast sample from a control or sALS patient. In the dendrograms shown (top and right), the length and subdivision of the branches display the relatedness of the expression of the genes (top) and the fibroblast samples (right). Heatmap colors represent relative mRNA expression as indicated in the color key: red indicates up-regulation, green indicates down-regulation, and black indicates no change. (**B**) Venn diagram showing overlap of our list of 277 DEGs with genes deregulated in the motor cortex of sALS patients described in our previous work [40].

Differential gene expression analysis between fibroblast lines of sALS patients and control subjects disclosed a total of 277 DEGs (336 probes), with 176 up-regulated and 160 down-regulated genes. A full list of DEGs is provided in Supplementary Table S1. Unsupervised hierarchical cluster analysis of samples and DEGs resulted in an overall separation of sALS fibroblast samples from controls on the basis of their expression patterns (Figure 1A). Among the 277 DEGs, 7 genes (*ALAD*, *ANXA2*, *DOC2B*, *DPP6*, *FBXO32*, *PARK2*, and *USP6NL*) are already known to be genetically associated with ALS. Moreover, beyond protein-coding genes, our analysis identified a number of non-coding transcripts, both pseudogenes and long non-coding RNAs, differentially expressed in sALS fibroblasts (*SNHG28*, *APRG1*, *FLJ30679*, *lnc-LONRF1-2*, *lnc-PPIAL4G-6*, *LINC02104*, *SOX5-AS1*, *DNAJC9-AS1*, *PAX8-AS1*, *PSMD5-AS1*, *PTPRD-AS1*, *PWAR5*, *ZNF702P*, *RPL14P4*, *EIF3FP2*, *RAC1P7*, *PPIAP42*, *NIP7P2*, *PRDX2P4*, *KRT8P36*, *YTHDF2P1*, *SNHG14)*, further supporting that the disruption of RNA metabolism may play a key role in ALS pathogenesis (Supplementary Table S1).

Of note, we found a substantial overlap between our DEG list and other gene expression studies in sALS fibroblasts (Supplementary Table S1) [46,47,59]. In particular, 215 (78%) of the 277 DEGs were detected as differentially expressed in sALS fibroblasts from an independent previously published study [59], with 117 of these genes showing a similar expression pattern (Supplementary Table S1 and Supplementary Figure S1). Moreover, some of these genes were also found statistically deregulated in other two transcriptomic studies on sALS fibroblasts [46,47], supporting the reliability of our molecular signature (Supplementary Table S1 and Supplementary Figure S1). A substantial overlap was also found between DEGs identified in this study and those previously found in the human motor cortex of sALS patients [40], supporting common disease-specific pathological mechanisms reverberating in both peripheral cells and brain tissue (Figure 1B, Supplementary Table S1).

### 3.2. Functional Enrichment Analysis Defines Key Factors and Processes Perturbed in sALS Fibroblasts

**Figure 2:**
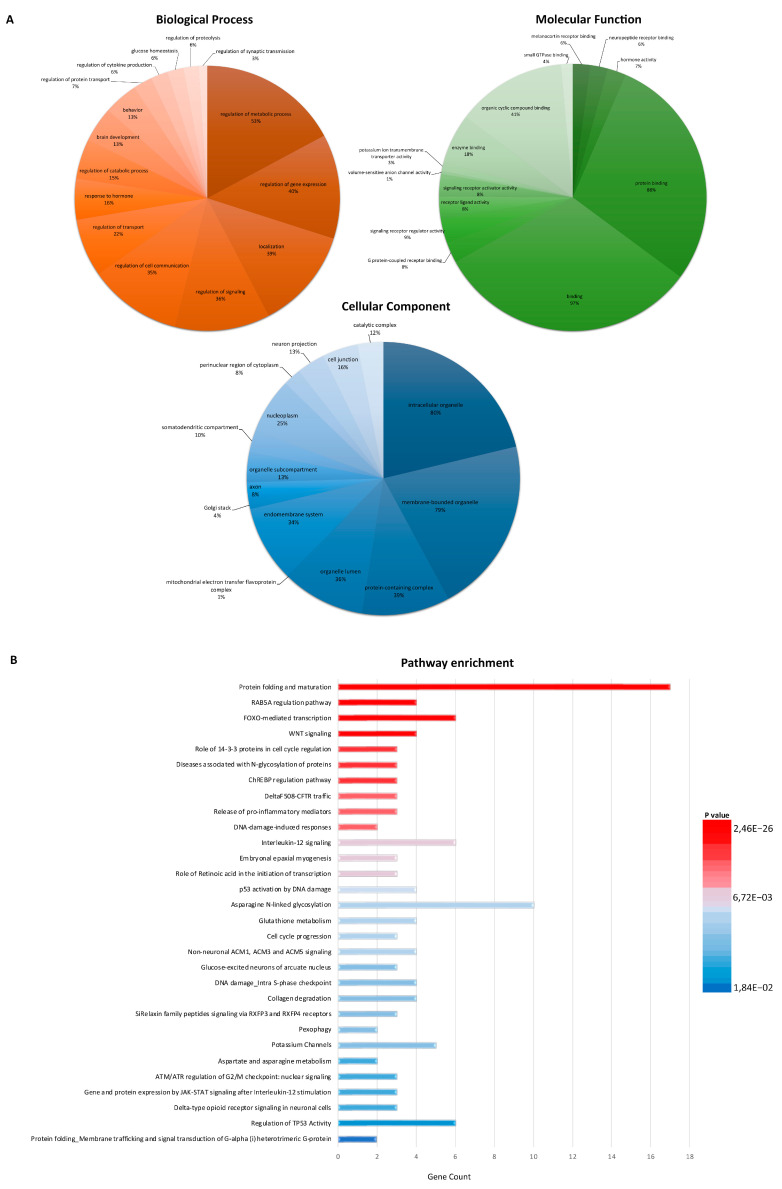
**Gene Ontology (GO) and pathway enrichment analysis of DEGs in sALS fibroblasts.** (**A**) Pie chart showing the gene ontology terms that are most represented in the 277 DEGs in sALS fibroblasts. The numbers are the percentage of genes in each category. (**B**) The top 30 functionally enriched pathways found in the analysis of DEGs in sALS fibroblasts vs. the control group. Gene ontologies were ranked by the number of genes related to the enriched pathway (gene count). The color of the bar denotes their significance.

To clarify the biological significance of the 277 DEGs, we performed a GO enrichment analysis (Figure 2A; Supplementary Tables S2–S4). This analysis identified a highly significant enrichment in biological processes related to the regulation of signaling, cell communication, metabolic/catabolic processes, synaptic transmission, response to stress, transport, and regulation of gene expression (Figure 2A; Supplementary Table S2). Neuropeptide receptor binding, hormone activity, and protein binding were among the most significantly enriched molecular functions in DEGs, while enriched GO CC terms included intracellular organelle, protein-containing complex, mitochondrial electron transfer flavoprotein complex, Golgi stack, and axon (Figure 2A; Supplementary Tables S3 and S4). Finally, a large number of pathways were identified as enriched in sALS fibroblasts, with the most significant pathways involving inflammation mediated by chemokine and cytokine signaling pathways, lipid metabolism, GABA receptor activation, cellular response to stress, mRNA splicing, FOXO-mediated transcription, neurotransmitter receptors, and post-synaptic signal transmission (Figure 2B; Supplementary Table S5).

### 3.3. PPI Network Analysis Reveals Important Hub Proteins and Sub-Network Modules

**Figure 3:**
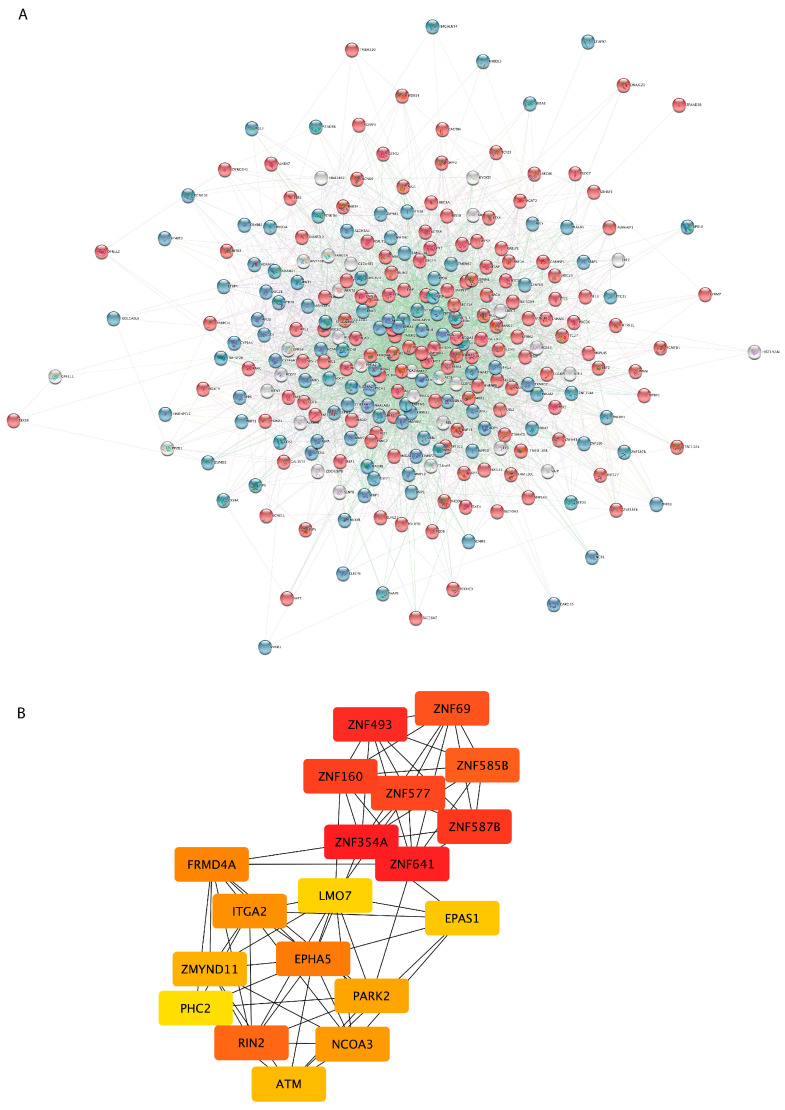
**PPI network construction and hub gene screening of DEGs in sALS fibroblasts.** (**A**) PPI network was constructed using the STRING Online Database and visualized by Cytoscape. Each node represents one gene/protein (n = 277), and the interaction between two nodes is defined as the edge (n = 3378). Node color is associated with the fold change: genes down-regulated in sALS fibroblasts vs. CTRL are colored in blue, while red nodes correspond to genes up-regulated in sALS fibroblasts vs. CTRL. (**B**) The network of the 20 hub genes is shown with red (high ranking) and yellow (low ranking) nodes, based on the ranking score in the cytoHubba plugin.

In order to reveal functional interactions among proteins coded by the 277 DEGs in sALS fibroblasts, a PPI network was constructed on the basis of the STRING database (Figure 3A). The resulting PPI network consisted of 277 nodes and 3378 edges. The top 20 nodes scoring the highest in MCC by the cytoHubba plugin were identified as hub genes in the network and might play crucial roles in ALS (Figure 3B). These hub genes were mainly related to the regulation of gene expression (*PHC2*, *EPAS1*, *ATM*, *ITGA2*, *ZNF577*, *ZNF354A*, *ZNF69*, *PARK2*, *NCOA3*, *ZNF493*, *ZMYND11*, *ZNF587B*, *ZNF160*, *LMO7*, *ZNF585B*, *ZNF641*, *EPHA5*), suggesting that dysregulation of this process may be the most relevant change in fibroblasts from sALS patients.

**Figure 4:**
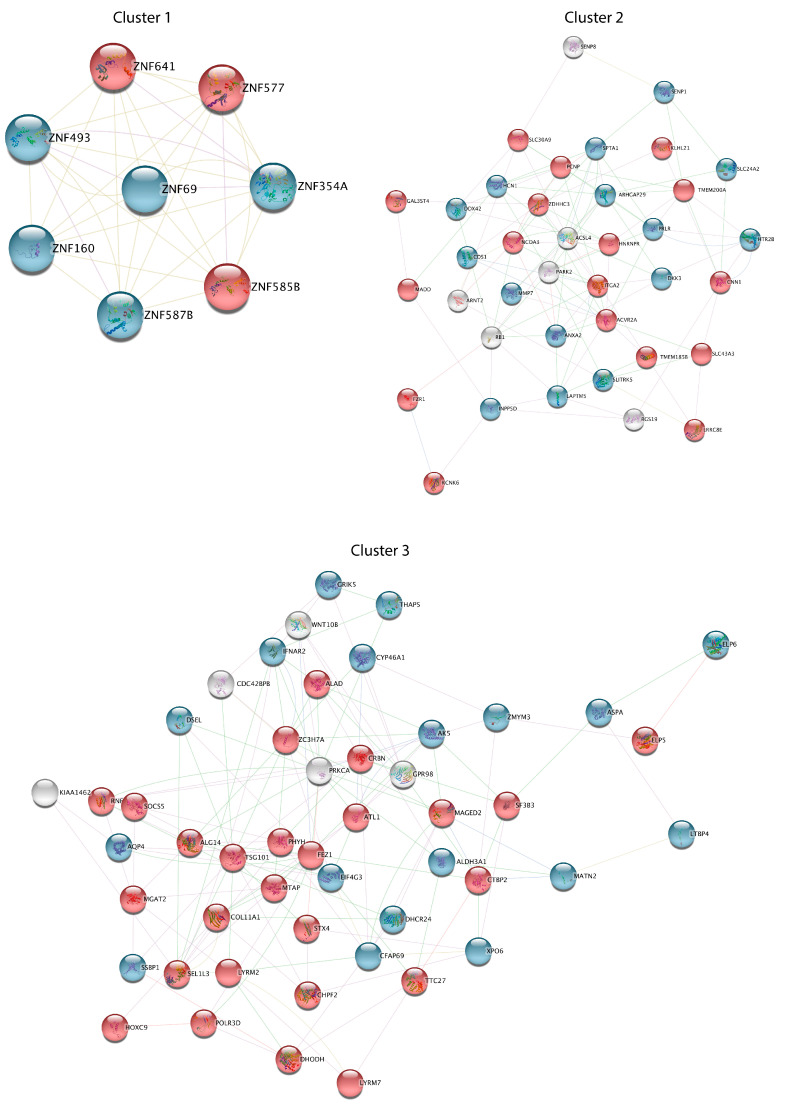
**Functional analysis of three DEG clusters in sALS fibroblasts.** Three significant interacting and functional clusters from the general PPI network were obtained by the MCODE plugin. Node color is associated with the fold change: genes down-regulated in sALS fibroblasts vs. CTRL are colored in blue, while red nodes correspond to genes up-regulated in sALS fibroblasts vs. CTRL.

Subsequently, the general PPI network was divided into subnetwork clusters to represent the main interacting and functional modules. Three functional clusters were identified by using the MCODE plugin and a score ≥ 5 as the cutoff (Figure 4). The most significant cluster, consisting of 8 nodes and 34 edges, includes genes encoding zinc finger proteins (*ZNF493*, *ZNF160*, *ZNF641*, *ZNF354A*, *ZNF585B*, and *ZNF577*), the largest transcription factor family in the human genome. Functional cluster 2, consisting of 38 nodes and 109 edges, includes genes involved in regulation of synaptic transmission, proteolysis, cell division, lipid metabolism, and signal transduction, while genes in cluster 3 (49 nodes and 139 edges) are significantly enriched in regulation of proteasomal catabolic processes, the Wnt signaling pathway, and response to cytokine stimulus.

### 3.4. Class Prediction Analysis

To test the reliability of our DEGs as a biomarker signature for discriminating ALS cases from controls, we carried out a supervised class prediction analysis applying a support vector machine with a linear kernel and a leave-one-out cross-validation (Table 3). Through this approach, we trained the prediction models with our ALS fibroblast dataset (*training set*) and then validated them in six previously published transcriptomic ALS studies (*test sets*), including gene expression data from skin and peripheral nerve-fibroblasts (GSE56808, GSE68240), whole blood (GSE11280, GSE112676, E-TABM-940), and post-mortem motor cortex (E-MTAB-2325) samples from controls and ALS patients [14,15,40,46,47,48] (Table 2 and Table 3). Our class prediction model correctly predicted 100% (12 of 12 correct calls) of the subjects in the *training set* and ≥73% in the *test sets* used for its validation (Table 3).

Moreover, in light of its potential utility as a diagnostic tool, we also evaluated whether the predictive accuracy and specificity of our prediction model were also confirmed by using a restricted list of deregulated genes. Of note, using the top 50 DEGs (the most 25 up- and down-regulated genes) between SALS fibroblasts and controls, the classifier algorithm also demonstrated that it correctly identified 100% of the subjects in the training data set and ≥75% of the subjects in the test data sets (Table 4).

## 4. Discussion

One paramount challenge in ALS is the lack of valid, reliable, and broadly usable biomarkers for an accurate diagnosis of this disorder, allowing effective therapeutic interventions. While approximately 70% of the genetic mutations that contribute to fALS have been identified, no genetic variations are found in the majority of sALS (85%), highlighting the complexity and genetic heterogeneity contributing to these sporadic cases.

During the last few years, several studies have demonstrated the value and utility of transcriptome profiling of post-mortem tissues in unravelling pathophysiological mechanisms underlying ALS and supporting the existence of a molecular taxonomy for this disease [9,19,20,21,22,23,24,25,26,27,28,29,30,31,32,33,34]. However, *post-mortem* analysis of ALS brain samples does not allow for evaluation of alterations occurring during the disease course; thus, it does not represent the optimal resource for biomarker discovery efforts. In this regard, peripheral cells, such as dermal skin-derived fibroblasts, may constitute a simple, viable, rapid, and cost-efficient translational model to investigate ALS, as they also recapitulate the genomic background of the patient, providing a rationale for utilizing them to find clinically useful diagnostic biomarkers of ALS [60,61,62,63].

In this study, we aimed to identify a fibroblast-related gene signature that would detect ALS accurately. Integrating transcriptome-wide analyses of patient-derived skin fibroblasts, we identified a gene expression signature that recapitulates previously determined dysregulated genes and pathways in the CNS of ALS patients and is able to distinguish sALS patients from control individuals. In particular, our analysis identified a total of 277 DEGs in sALS patients when compared to controls, which were predominantly involved in pathways previously associated with ALS pathogenesis, including RNA processing, response to stress, transport, and intracellular signaling [2,45,64] (Figure 1 and Figure 2; Supplementary Tables S2–S5). Of note, we found a significant overlap between the list of DEGs in sALS fibroblasts and those previously identified in the motor cortex of sALS patients [40], suggesting the ability of our peripheral gene expression signature to recapitulate characteristics of ALS pathology and, thus, further sustaining its potential diagnostic utility (Figure 1B, Supplementary Table S1).

Some of the identified DEGs have been associated with ALS, including three genes up-regulated (*ALAD*, *FBXO32*, and *USP6NL*) or down-regulated (*ANXA2*, *DOC2B*, *DPP6,* and *PARK2)* in sALS fibroblasts (Supplementary Table S1). *ALAD* encodes an enzyme involved in oxidative stress that influences susceptibility to lead exposure and contributes to MND risk [64,65,66]. The significant up-regulation of *FBXO32*, previously observed in the skeletal muscles of ALS transgenic mice, correlates with muscle atrophy during disease progression [67,68]. According to previous studies, we observed the down-regulated expression of *DOC2B*, a gene involved in Ca^2+^-dependent intracellular vesicle trafficking and synaptic function, and *ANXA2*, which encodes a member of the annexin family involved in calcium-homeostasis and intracellular calcium-regulated pathways [59,69,70]. Decreased expression of *DPP6*, whose genetic alterations have been associated with susceptibility to ALS and that is involved in membrane excitability, was previously reported in both CNS tissues of sALS patients as well as in other in vitro ALS models [40,71,72,73,74]. Dysregulated expression of the Parkinson’s disease gene *PARK2* was previously found in the spinal cord and motor cortex samples of sALS patients, as well as in ALS animal models, supporting this gene as a disease modifier in ALS pathogenesis [75,76,77].

Our PPI network analysis revealed several key hub genes that may have potential roles in sALS, confirming the implication of cell communication, metabolic/catabolic processes, synaptic transmission, oxidative stress, transport, and transcriptional regulation in sALS pathology (Figure 3 and Figure 4). In particular, interactome analysis identified a cluster of hub genes involved in the regulation of gene expression (*ATM*, *EPHA5*, *EPAS1*, *ITGA2*, *LMO7*, *NCOA3*, *PHC2*, *PARK2*, *ZNF577*, *ZNF354A*, *ZNF69*, *ZNF493*, *ZMYND11*, *ZNF587B*, *ZNF160*, *ZNF585B*, *ZNF641*), supporting dysfunctions in the RNA metabolism process and transcriptional machinery as key processes in the pathogenesis of ALS [78,79,80,81,82] (Figure 3B). Among these genes, of particular interest are the zinc finger proteins involved in cluster 1 (*ZNF493*, *ZNF160*, *ZNF641*, *ZNF354A*, *ZNF585B*, and *ZNF577*) (Figure 3B and Figure 4). Indeed, multiple transcription factors belonging to this family were found dysregulated in both human patients with ALS and animal models and may contribute to the pathogenic phenotype by interacting with multiple RNA-binding proteins, including the ALS-associated proteins FUS and TDP43, and altering DNA damage repair processes [83,84,85,86]. In addition, the use of zinc finger protein transcription factors has been recently investigated for the development of an ALS gene therapy [86]. The down-regulated expression of *EPHA5* observed in sALS fibroblasts is in line with previous findings implicating ephrin-A5 as a modifier of disease progression in both ALS patients and animal models [87]. Of note, despite the fact that our platform is not ideal for comprehensively investigating the role of non-coding genes, our analysis revealed a number of deregulated pseudogenes and antisense long non-coding RNA in sALS fibroblasts, further supporting the important contribution of defective RNA metabolism in the pathogenesis of ALS and the potential role of non-coding RNA transcripts as diagnostic biomarkers [82,88,89,90,91,92] (Supplementary Table S1). Among these, the differential expression of *PSMD5-AS1*, encoding PSMD5 antisense RNA 1, was previously found in induced pluripotent stem cell (iPSC)-derived motor neurons from patients with ALS [93]. In sALS fibroblasts, we also found increased expression of the PAX8 antisense RNA1 (*PAX8-AS1*), a long non-coding RNA that is linked to cell cycle control and metabolic processes previously reported to be associated with different neurodegenerative diseases, including Parkinson’s disease and Huntington’s disease [94,95].

The functional analysis of down-regulated genes in clusters 2 and 3 revealed their involvement in transmembrane transport (*AQP4*, *ANXA2*, *GRIK5*, *HCN1*, *HTR2B*, *SLC24A2*), extracellular matrix organization (*ANXA2*, *LTBP4*, *MATN2*, *MMP7)*, and synaptic transmission (*GRIK5*, *HTR2B*, *SLC24A2*), while the up-regulated genes in these clusters were mainly enriched in metabolic processes (*ACVR2A*, *ALAD*, *ALG14*, *FZR1*, *HOXC9*, *MGAT2*, *RNF14*, *SOCS5*, *ZDHHC3*) and protein ubiquitination (*CRBN*, *PCNP*, *RNF14*), supporting the pathogenic role of these processes in ALS [96,97,98,99,100,101,102,103,104,105,106,107] (Figure 4). Of interest, decreased expression of *SLC24A2*, *GRIK5*, and *HCN1*, three genes involved in the regulation of neuronal excitability and modulation of synaptic transmission and plasticity, was previously reported in motor neurons of ALS patients and animal models [108,109,110].

In an effort to examine the reliability of our transcriptomic signature as a potential molecular classifier, we tested the predictive power of the 277-gene signature to distinguish sALS patients from controls in our training dataset and in six independent test datasets obtained from different ALS tissue/cell types (Table 3). Our transcriptome-based classification model showed consistent performance in discriminating between ALS patients and healthy controls, with high rates of sensitivity, specificity, and accuracy in the training and test sets (Table 3). Similar yields in terms of sensitivity, specificity, and accuracy rate were also obtained by using the list of the top 25 up- and down-regulated genes among the 277 identified DEGs (Table 4), suggesting that these gene expression signatures may be used as potential biomarkers for the development of a transcriptome-based diagnostic test for ALS. Of note, our estimates compared well to those previously reported for ALS blood gene expression signatures (reporting an accuracy~87%, sensitivity~86%, and specificity~87%), sustaining the potential utility of our transcriptomic signature for ALS diagnostics [14,15] (Table 3).

## 5. Conclusions

The use of multigene mRNA-based diagnostic assays is already included in the clinical guidelines for different pathologies, but their use in neurodegenerative diseases has been complicated by the inaccessibility of the diseased tissue. Our findings showed that a transcriptome-based signature obtained from ALS fibroblasts, an easily accessible sample type, recapitulates characteristics of brain and blood pathology. Despite the relatively small number of samples analyzed in this study, to our knowledge, this work provides the largest gene expression profiling in sALS fibroblasts to date and points to the promise of using a transcriptional signature in peripheral cells as a suitable diagnostic tool for ALS diagnostics. It remains to be determined how early these alterations can be detected. Further studies are thus needed to evaluate the potential clinical validity and utility of this signature in clinical practice, together with its temporal performance and differential diagnosis capacity.

## Figures and Tables

**Table 1:** Clinical and demographic characteristics of the sALS patients and the controls.

Variable	ALS	Healthy Controls	*p*
	(n = 9)	(n = 3)	
Age at onset	63 (42–77)	N.A.	
Age at skin biopsy	64 (47–79)	64 (56–72)	0.85 *
Sex (M/F)	3/6	1/2	0.90 **
ΔFS	0.52 (0.39–1.11)		
Site of onset (n,%)			
Spinal	6 (66.6)		
Bulbar	3 (33.4)		

Data are expressed as medians with interquartile ranges (IQR). * Mann-Whitney Rank Sum Test; ** chi-square.

**Table 2:** Transcriptomic datasets used as test data sets in class prediction analysis.

Accession Number	Repository	Platform	Sample Type	Number of Samples (ALS/Controls)	References
GSE56808	GEO	Affymetrix Human Genome U133 Plus 2.0 Array	Fibroblasts	12 (6/6)	[47]
GSE68240	GEO	Agilent-028004 SurePrint G3 Human GE 8x60 K Microarray	Fibroblasts	6 (3/3)	[46]
GSE112680	GEO	Illumina HumanHT-12 V4.0 expression beadchip	Whole blood	301 (164/137)	[15]
GSE112676	GEO	Illumina HumanHT-12 V3.0 expression beadchip	Whole blood	741 (233/508)	[14,15]
E-TABM-940	ArrayExpress	Affymetrix GeneChip Human Genome U133 Plus 2.0	Whole blood	85 (57/28)	[48]
E-MTAB-2325	ArrayExpress	Agilent-014850 Whole Human Genome Microarray 4x44 K	Motor cortex	41 (31/10)	[40]

**Table 3:** Summary of the prediction results for the 277 DEG transcriptome signature in training and test/validation sets.

	Training Set (GSE233881)		Test Set 1 (GSE56808)		Test Set 2 (GSE68240)		Test Set 3 (GSE112680)		Test Set 4 (GSE112676)		Test Set 5 (E-TABM-940)		Test Set 6 (E-MTAB-2325)	
	ALS (n = 9)	CTRL (n = 3)	ALS (n = 6)	CTRL (n = 6)	ALS (n = 3)	CTRL (n = 3)	ALS (n = 164)	CTRL (n = 137)	ALS (n = 233)	CTRL (n = 508)	ALS (n = 57)	CTRL (n = 28)	ALS (n = 31)	CTRL (n = 10)
Correct number of patients	9	3	5	5	2	3	144	113	142	399	55	19	28	6
Incorrect number of patients	0	0	1	1	1	0	20	24	91	109	2	9	3	4
Accuracy *	100%		83%		83%		85%		73%		87%		83%	
Sensitivity **	100%		83%		67%		88%		61%		96%		90%	
Specificity ***	100%		83%		100%		82%		78%		68%		60%	

TruePositive (TP): the number of cases correctly identified as patients. True Negative (TN): the number of cases correctly identified as healthy. False Positive (FP): the number of cases incorrectly identified as patients. False negative (FN): the number of cases incorrectly identified as healthy cases. * Accuracy = TP + TN/TP + TN + FP + FN; ** Sensitivity (%) = TP/(TP + FN); *** Specificity (%) = TN/(TN + FN).

**Table 4:** Summary of the prediction results for the top 50 DEG transcriptome signatures in training and test/validation sets.

	Training Set (GSE233881)		Test Set 1 (GSE56808)		Test Set 2 (GSE68240)		Test Set 3 (GSE112680)		Test Set 4 (GSE112676)		Test Set 5 (E-TABM-940)		Test Set 6 (E-MTAB-2325)	
	ALS (n = 9)	CTRL (n = 3)	ALS (n = 6)	CTRL (n = 6)	ALS (n = 3)	CTRL (n = 3)	ALS (n = 164)	CTRL (n = 137)	ALS (n = 233)	CTRL (n = 508)	ALS (n = 57)	CTRL (n = 28)	ALS (n = 31)	CTRL (n = 10)
Correct number of patients	9	3	5	5	2	3	128	96	115	437	50	20	26	6
Incorrect number of patients	0	0	1	1	1	0	36	41	118	71	7	8	5	4
Accuracy *	100%		83%		83%		75%		75%		83%		83%	
Sensitivity **	100%		83%		67%		78%		50%		88%		84%	
Specificity ***	100%		83%		100%		70%		86%		72%		60%	

TruePositive (TP): the number of cases correctly identified as patients. True Negative (TN): the number of cases correctly identified as healthy. False Positive (FP): the number of cases incorrectly identified as patients. False negative (FN): the number of cases incorrectly identified as healthy. * Accuracy = TP + TN/TP + TN + FP + FN; ** Sensitivity (%) = TP/(TP + FN); *** Specificity (%) = TN/(TN + FN).

## Data Availability

Data are contained within the Supplementary Materials. Microarray raw data were deposited in NCBI’s GEO with the accession number GSE233881.

## Supplementary Materials

The following supporting information can be downloaded at: https://www.mdpi.com/article/10.3390/cells12141884/s1. Supplementary Figure S1. Heatmaps showing the overlap between expression levels of DEGs detected in our sALS fibroblast cohort and other gene expression studies in sALS fibroblasts. Supplementary Table S1. List of differentially expressed genes in sALS fibroblasts vs. control individuals. Supplementary Table S2. List of Gene Ontology-Biological Processes terms enriched in 277 genes DE in sALS fibroblasts vs. Ctrl. Supplementary Table S3. List of Gene Ontology-Molecular Function terms enriched in 277 genes DE in sALS fibroblasts vs. Ctrl. Supplementary Table S4. List of Gene Ontology-Cellular Component terms enriched in 277 genes DE in sALS fibroblasts vs. Ctrl. Supplementary Table S5. List of biological pathways enriched in 277 genes DE in sALS fibroblasts vs. Ctrl.

## Abbreviations

ALS = Amyotrophic lateral sclerosis; sALS = sporadic ALS; fALS = familial ALS; CNS = central nervous system; SOD1 = superoxide dismutase type 1; ANG = angiogenin; FUS = fused in sarcoma; TARDBP = TAR DNA binding protein; DMEM = Dulbecco’s Modified Eagle Medium; RIN = RNA integrity score; cDNA = complementary DNA; DEG = differentially expressed gene; Cy3 = Cyanine-3; GEO = Gene Expression Omnibus; FDR = False Discovery Rate; SVM = Support Vector Machines; CTRL = Control; GO = gene ontology; BP = biological processes; CC = cellular component; MF = molecular function; PPI = protein-protein interaction; STRING = Search Tool for the Retrieval of Interacting Genes/Proteins; MCC = maximum correlation criterion; MCODE = Molecular Complex Detection; ALAD = Aminolevulinate Dehydratase; ANXA2 = Annexin A2; DOC2B = double C2 domain beta; DPP6 = dipeptidyl peptidase like 6; FBXO32 = F-box protein 32; PARK2 = parkin RBR E3 ubiquitin protein ligase; USP6NL = USP6 N-terminal like; GABA = gamma-aminobutyric acid; FOXO = forkhead box O; PHC2 = polyhomeotic homolog 2; EPAS1 = endothelial PAS domain protein 1; ATM = Ataxia-telangiectasia mutated; ITGA2 = integrin subunit alpha 2; ZNF577 = Zinc Finger Protein 577; ZNF354A = Zinc Finger Protein 354A; ZNF69 = Zinc Finger Protein 69; ZNF493 = Zinc Finger Protein 493; ZNF160 = Zinc Finger Protein 160; ZNF587B = Zinc Finger Protein 587B; ZNF641 = Zinc Finger Protein 641; ZNF585B = Zinc Finger Protein 585B; MND = motor neuron disease; NCOA3 = nuclear receptor coactivator 3; AQP4 = aquaporin 4; ZMYND11 = zinc finger MYND-type containing 11; LMO7 = LIM only protein 7; EPHA5 = ephrin receptor A5; ITGA2 = integrin alpha 2; LMO7 = LIM domain only protein-7; PHC2 = polyhomeotic homolog 2; PCNP = PEST proteolytic signal containing nuclear protein; CRBN = cereblon; GRIK5 = glutamate ionotropic receptor kainate type subunit 5; HCN1 = hyperpolarization activated cyclic nucleotide gated potassium channel 1; HTR2B = 5-hydroxytryptamine (serotonin) receptor 2B; SLC24A2 = solute carrier family 24 member 2; LTBP4 = latent transforming growth factor beta binding protein 4; MATN2 = matrilin 2; MMP7 = matrix metalloproteinase 7; ACVR2A = activin A receptor type 2A; ALG14 = asparagine-linked glycosylation 14; FZR1 = fizzy and cell division cycle 20 related 1; HOXC9 = homeobox C9; MGAT2 = mannoside acetylglucosaminyltransferase 2; RNF14 = ring finger protein 14; SOCS5 = Suppressor Of Cytokine Signaling 5; ZDHHC3 = Zinc Finger DHHC-Type Palmitoyltransferase 3.
