# Supplementary material for: A Diagnostic Gene-Expression Signature in Fibroblasts of Amyotrophic Lateral Sclerosis
Source: Cells. 2023 Jul 18;12(14):1884. doi: 10.3390/cells12141884 (PMC10378077; doi:10.3390/cells12141884)
Supplement: Supplementary file 1 [file cells-12-01884-s001.zip › Supplementary Figure S1.pdf]

Supplementary Figure S1. Heatmaps showing the overlap between expression levels (fold change ratio) of DEGs detected in our sALS fibroblast cohort with other gene expression studies in sALS fibroblasts. Down-regulation is coloured in shades of green, while up-regulation is coloured in shades of red.

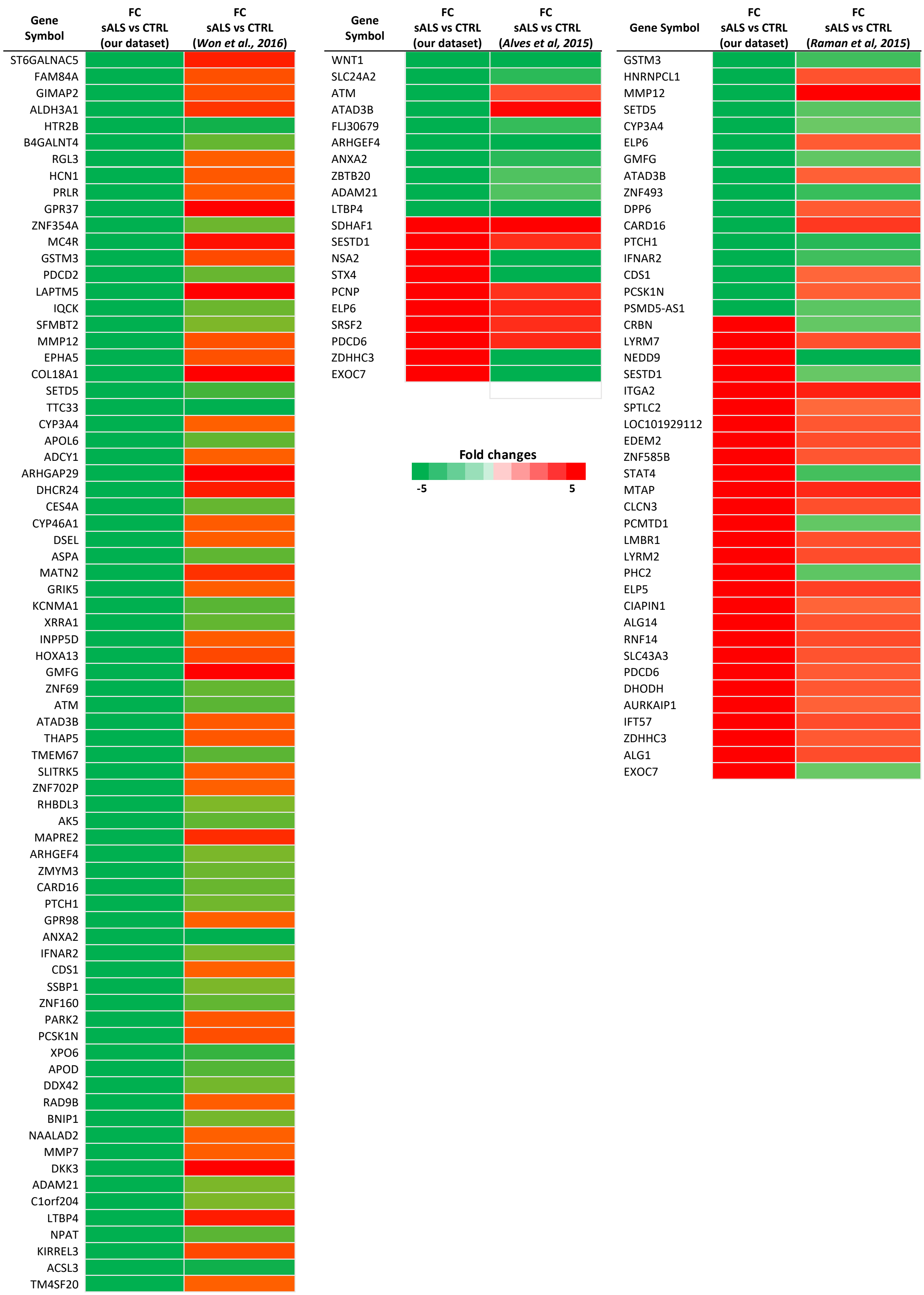

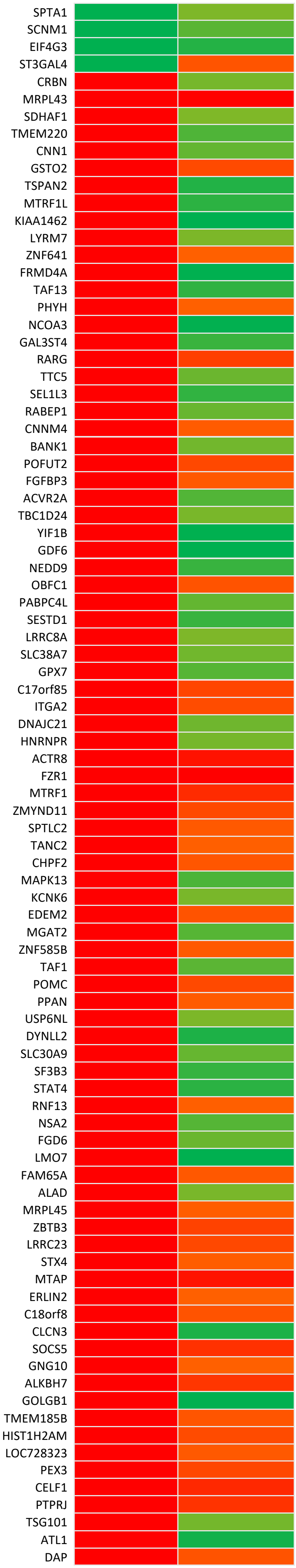
SPTA1
SCNM1
EIF4G3
ST3GAL4
CRBN
MRPL43
SDHAF1
TMEM220
CNN1
GSTO2
TSPAN2
MTRF1L
KIAA1462
LYRM7
ZNF641
FRMD4A
TAF13
PHYH
NCOA3
GAL3ST4
RARG
TTC5
SEL1L3
RABEP1
CNNM4
BANK1
POFUT2
FGFBP3
ACVR2A
TBC1D24
YIF1B
GDF6
NEDD9
OBFC1
PABPC4L
SESTD1
LRRC8A
SLC38A7
GPX7
C17orf85
ITGA2
DNAJC21
HNRNPR
ACTR8
FZR1
MTRF1
ZMYND11
SPTLC2
TANC2
CHPF2
MAPK13
KCNK6
EDEM2
MGAT2
ZNF585B
TAF1
POMC
PPAN
USP6NL
DYNLL2
SLC30A9
SF3B3
STAT4
RNF13
NSA2
FGD6
LMO7
FAM65A
ALAD
MRPL45
ZBTB3
LRRC23
STX4
MTAP
ERLIN2
C18orf8
CLCN3
SOCS5
GNG10
ALKBH7
GOLGB1
TMEM185B
HIST1H2AM
LOC728323
PEX3
CELF1
PTPRJ
TSG101
ATL1
DAP

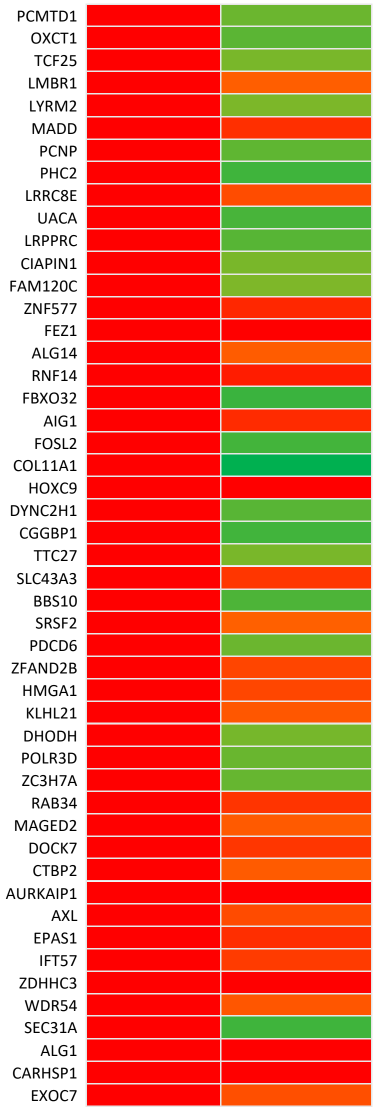
PCMTD1
OXCT1
TCF25
LMBR1
LYRM2
MADD
PCNP
PHC2
LRRC8E
UACA
LRPPRC
CIAPIN1
FAM120C
ZNF577
FEZ1
ALG14
RNF14
FBXO32
AIG1
FOSL2
COL11A1
HOXC9
DYNC2H1
CGGBP1
TTC27
SLC43A3
BBS10
SRSF2
PDCD6
ZFAND2B
HMGA1
KLHL21
DHODH
POLR3D
ZC3H7A
RAB34
MAGED2
DOCK7
CTBP2
AURKAIP1
AXL
EPAS1
IFT57
ZDHHC3
WDR54
SEC31A
ALG1
CARHSP1
EXOC7
